# The effect of working in an auto factory on functional constipation and bowel habits 

**Published:** 2019

**Authors:** Hadis Najafimehr, Hosein Yadegari, Hamed Taherinejad, Khosrow Manhoie, Seyed Ramin Rasooli, Abbas Moradi, Mohamad Javad Akbariju, Hosein Mohseni, Sabah Ghadimi, Hamid Mohaghegh Shalmani

**Affiliations:** 1 *Gastroenterology and Liver Diseases Research Center, Research Institute for Gastroenterology and Liver Diseases, Shahid Beheshti University of Medical Sciences, Tehran, Iran*; 2 *Basic and Molecular Epidemiology of Gastrointestinal Disorders Research Center, Research Institute for Gastroenterology and Liver Diseases, Shahid Beheshti University of Medical Sciences, Tehran, Iran*; 3 *Department of Occupational Health & Safety, Company of SAIPA Automotive Corporation, Tehran, Iran*; 4 *Department of Administrative Management, Company of SAIPA Automotive Corporation, Tehran, Iran *; 5 *Faculty of Medicine, * *Shahid Beheshti University of Medical Sciences, Tehran, Iran*

**Keywords:** Bowel movement, Constipation, Work shift, Auto factory

## Abstract

**Aim:**

The aim of the present study was to evaluate the factors associated with functional constipation (FC) and to determine a normal range of bowel movement (BM) in an Iranian Auto factory’s workers.

**Background:**

The digestive system may be affected by workplace conditions. Some occupational conditions can affect the bowel habit and FC.

**Methods:**

In this cross-sectional study, 3590 workers who worked in Tehran suburb in 2017 were evaluated. The workers worked in morning or rotatory shifts and in the official and non-official sections. In addition to demographic and stool frequency questions, workers were asked to complete the Rome IV Questionnaire.

**Results:**

The normal range of BM frequency was determined between one and three per day. The BM frequency had a significant association with age (P=0.002), marital status (P=0.024), education (P=0.011), exposure to chemical materials (P<0.001), and work section (P<0.001). The total prevalence of FC was 9.7% which was greater among rotatory shift working than among only morning shift workers (10% vs 6%; P=0.02). Independent factors associated with FC were found as age (for 30- 40 years old: OR=1.88; 95% CI (1.20, 3.03) and for ≥41 years old: OR=1.91; 95% CI (1.12,3.17)), smoking (OR=1.52; 95% CI (1.20,1.93)) and work section (for Paint section: OR=0.33; 95% CI (0.12,0.87), for montage section: OR=0.44; 95% CI (0.18,1.10), for press & platform section: OR=0.12; 95% CI (0.05,0.37)).

**Conclusion:**

Occupational condition may make a difference in bowel habit. Rotatory shift, official working, and smoking may increase the risk of constipation.

## Introduction

 The frequency of bowel movement (BM) is defined as” the number of daily stool defecation for a person”. There is no general instruction for BM frequency definition as each person may have an individual number of stool frequency per day (1). Bowel habit be affected by food habitation and fiber consumption as well as by geographical and cultural factors. Further, BM frequency varies from person to person (2, 3). A high frequent BM is known as BM- Diarrhea while the low frequent is known as constipation. High frequent BM may be due to some factors such as pancreatic issues, food allergies and irritable bowel syndrome and even due to some cancers and may appear with some symptoms such as urgent need to pass stool, bloating, or fever (4, 5). Low frequent (Infrequent) BM occurs due to bowel obstruction, diminished motility, stress, and some cancers. Infrequent BM leads to some effects such as anorexia, cardiovascular disease, and inflammation (6). Both frequent and infrequent BM are problematic for a normal person. A normal person should have a normal BM frequency. Studies have shown that most normal people have one BM per day which can be considered normal (7-9). 

Nowadays, in many regions of the world, the modern lifestyle has altered constipation to a common gastrointestinal problem (10-12). The constipation symptoms include hard stool, incomplete defecation, bloating, and pain which is also costly for the healthcare system. The prevalence of self- report constipation in some countries has been reported up to 28% per population (11, 13). The constipation may be affected by some factors such as age, sex, socioeconomics, and education (14). In some researches for a random major population, less than 3 BM per week was considered as a sign of constipation (15), while in some other studies, less than 4 times per week was an indicator of constipation (16). Therefore, it is hardly possible to define a normal standard BM universally. 

A suitable definition for normal BM can be a helpful means for diagnosis and treatment of constipation and diarrhea. Meanwhile, people may have a different bowel habit given their special environmental or occupational situation (17). Previous studies concerning the factors associated with the bowel habit and especially the normal range of BM, have been conducted on a western random population (10, 18, 19). On the other hand, there is a lack of such studies for the Iranian population employed in different sectors. To address this gap, the present study provided a normal BM definition for a large population of Auto factory’s workers. It also evaluated the association between functional constipation (FC) and demographic variables as well as job features. 

## Methods

**Design and Questionnaire**

This study is a part of a gastrointestinal case finding survey performed on workers in SAIPA Auto factory, located in Tehran suburb, in 2017. In this cross-sectional survey, all workers were invited to participate in the study; most workers were male, so only male workers were enrolled in the study. Inclusion criteria included being a man, working in the SAIPA factory for at least six months, and signing a consent form to participate in the study. Workers of different sections of the factory, including Administrative and Support (A), Paint (P), Montag (M), Body (B), and Press & Platform (P&P) were evaluated. According to work times, they were divided into two categories: day shift between 7:00 and 15:30, and rotatory shift here workers’ shift changes each week in three shifts of day shift (between 7:00 and 15:30), evening shift (between 16:00 and 24:00), and night shift (between 24:00 and 07:00). Workers usually worked from Saturday to Thursday with every Friday off. The working shift in the last three months was considered as the workers’ shift time. 

A questionnaire, including demographic data, habits, gastrointestinal manifestations within the last 3 months, past medical history, family history, and drug history was completed for each worker via face to face interview. Gastrointestinal (GI) symptoms, including abdominal pain/discomfort, constipation, acute diarrhea, chronic diarrhea, bloating/flatulence, heartburn/acid regurgitation, diminished appetite, nausea/vomiting, bloody or black stool (melena), anorexia/weight loss, and difficulty of swallowing were asked.

**Table1:** Diagnostic Criteria for Functional Constipation

1. Must include 2 or more of the following:
a. Straining during more than one-fourth (25%) of defecations
b. Lumpy or hard stools (BSFS 1−2) more than one-fourth (25%) of defecations
c. Sensation of incomplete evacuation more than one-fourth (25%) of defecations
d. Sensation of anorectal obstruction/blockage more than one-fourth (25%) of defecations
e. Manual maneuvers to facilitate more than one fourth (25%) of defecations (e.g., digital evacuation, support of the pelvic floor)
f. Fewer than 3 spontaneous BMs per week
2. Loose stools are rarely present without the use of laxatives
3. Insufficient criteria for irritable bowel syndrome
a Criteria fulfilled for the last 3 months with symptom onset at least 6 months prior to diagnosis
b For research studies, patients meeting criteria for OIC should not be given a diagnosis of FC as it is difficult to distinguish
between opioid side effects and other causes of constipation. However, clinicians recognize that these 2 conditions might overlap.

All questionnaires were evaluated by a physician. Those who required further evaluation were asked for laboratory tests and or imaging; if required, they were referred to a gastroenterologist. Note that GI symptoms and GI diagnoses were dependent variables.

We used data regarding BM and constipation. The frequency of BM was asked from workers as: “what is the frequency of BM (without using laxatives)? Once every day Once every …. ..…times a day”. All data ware converted to daily BM and reported for each subject.

Among those who had complaint for constipation, ROME IV criterion was used to assess FC (Table 1) (20). The validity and reliability of the Persian version of ROME III criteria had already been tested in a pilot study. For validity study, language, content, concurrence, and construct validities were examined with the Cronbach’s alpha coefficient values being above 0.7 for all of the major symptoms included in the instrument (21). ROME IV and III criteria are very similar and have slight differences, though the ROME IV are slightly more precise than the version III.

**Statistical analysis**

Descriptive statistics were used including median ± interquartile range (IQR) for non- normal variables along with number and percentage for discrete variables. Also, the percentiles of BM distribution were shown. In evaluating the association between BM frequency with demographic as well as occupational features variables, Mann-Whitney and Kruskal- Wallis tests were employed. Pearson’s chi-square was performed to test the independence of classification variables. In order to capture the association between constipation and categorical variables, logistic regression was done with odds ratio (OR) and 95 % confidence interval reported. For statistical tests, the significance level was considered as 0.05. All analyses were performed using SPSS (version 21) software. 

## Results

**Demographic data**

Of 5910 invited workers, 3590 participated in the study (response rate: 60.7%). All workers were men with a mean age of 36.46 ± 4.90 years and mean Body Mass Index (BMI) of 25.90 ± 2.81 kg/m2. Table 2 reports the demographic data of the studied subjects. 

**Table 2:** Demographic data and main characteristics of all workers

Number of workers (%)	Variable	
	Age	
357 (10.2)	20-30	
2539 (70.7)	31-40	
596 (16.5)	>=41	
	BMI^*^	
8 (0.2)	<18.5	
510 (14.2)	18.5-22.99	
2977 (82.9)	>=23	
	Smoking	
1167 (32.5)	Yes	
2423 (67.5)	No	
	Alcohol consumption	
208 (5.8)	Yes	
3382 (94.2)	No	
	Expose to chemical materials	
990 (27.6)	Yes	
2600 (72.4)	No	
	Work shift	
379 (10.6)	Only morning	
3170 (88.3)	Rotatory	
	Marital status	
3273 (91.2)	Married	
312 (8.7)	Single or divorced	
	Education status	
134 (3.7)	Under diploma	
2222 (61.9)	Diploma	
1234 (34.4)	Upper diploma	
	Work section	
31 (0.9)	A^+^	
820 (22.8)	P^++^	
1466 (40.80)	M^+++^	
992 (27.6)	B^++++^	
281 (7.80)	P&P^+++++^	

*: body mass index; +: Administrative and supporting; ++: Paint; +++: Montag; ++++: Body; +++++: press & platform

In the factory, there were five kinds of work section: Administrative and Support (A), Paint (P), Montage (M), Body (B), and Press & Platform (P&P). Also, there were two types of work shift: only morning and rotatory. From 3590 workers, 0.9 % worked in section A, 22.8% in section P, 40.80% in section M, 27.6 % in section B, and 7.80 % in section P&P. The only morning workers were 10.7 % while 89.3 % worked in the rotatory shift. 

**Bowel movement**

The median frequency of BM was 2.00 ± 1.00 per day. The descriptive statistics for BMs per day in the Auto factory’s workers are shown in Table 3. 

**Table 3:** Daily bowel movement statistics in the Auto factory’s workers

Mean		1.80
Median		2.00
Mode		2
Std. Deviation		0.93
	2.5 Percentile	0.00
	3 Percentile	0.00
	5 Percentile	1.00
	10 Percentile	1.00
	25 Percentile	1.00
	75 Percentile	2.00
	80 Percentile	2.00
	90 Percentile	3.00
	95 Percentile	4.00
	97 Percentile	4.00
	97.5 Percentile	4.00

According to 5th and 95th centiles, the normal range of BM was between one and three per day. Among workers, 1342 (37.4 %) had regular one BM per day, while 184 (5.2 %) had equal to or greater than 4 BM per day. A total of 126 (3.5 %) workers had no BM per day and 9 (0.25 %) workers had less than three per week. Thus, 91.3% were within the normal range based on our evidence. 

The association between frequency of BM and age (P- value= 0.002), marital status (P- value= 0.024), education (P- value= 0.011), exposure to chemical materials (P- value< 0.001), and work section (P- value< 0.001) was statistically significant while BM frequency had no association with BMI, smoking, alcohol consumption, and work shift (all P- value > 0.05). 

**Functional constipation**

According to Rome-IV criteria, 347 subjects (9.7 %) had FC and their mean BM frequency was 1.63 ± 0.83; for other subjects who were not constipated, this mean was 1.89 ± 0.88. The prevalence of FC was increased with age; among 20- 30 and 31- 40, and more than 41 years old, it was 11 %, 6 %, 10 % respectively (P= 0.034). The FC prevalence was higher among smokers than in non-smokers: 12 % vs 8 % (P < 0.001). For workers who had exposure to chemicals, the prevalence was more than the others: 14 % vs 8 % (P < 0.001). The workers working in the rotatory shift had a higher constipation prevalence than the only morning shift: 10 % vs 6 % (P= 0.025). FC prevalence among section A (official section) workers was more than the other sections: in section A it was 26 %, in section P 13 %, in section M 13 %, in section B 2 % and in section P & P 4 % (P < 0.001) (Table 4). 

In order to investigate the factors associated with constipation, the logistic regression model was used. In the univariate logistic model, each of the variables including age, smoking, work shift, work section, and exposure to chemical materials were risk factors for constipation (P= 0.043, P < 0.001, P < 0.001, P= 0.025, P < 0.001, respectively). In the multivariate state, by controlling the mentioned significant risk factors, the chance of constipation increased with age (for 31- 40 years old: OR= 1.88; 95 % CI (1.20, 3.03) and for ≥ 41 years old: OR= 1.91; 95 % CI (1.12, 3.17)). The smokers had a higher chance of being affected in contrast to non-smokers (OR= 1.52; 95 % CI (1.20, 1.93)). Also, the chance of constipation in section A was more than in other sections (for section P: OR= 0.33; 95 % CI (0.12, 0.87), for section B: OR= 0.06; 95 % CI (0.02, 0.16) and for section P & P: OR= 0.12; 95 % CI (0.05, 0.37)) (Table 4).

## Discussion

There is a sparse data on the BM pattern of healthy employees working in special occupations. Information about normal BM in a population with a particular condition may be helpful for constipation or diarrhea treatment. This study evaluated bowel movement frequency and also FC sources in active young male groups, whereby we found that 94.73 % had BM between one per day and 3 per week and 9.7 % had FC. 

The first objective of the present study was to identify a normal BM range to determine the cut-off points for diagnosis of diarrhea or constipation in an active Iranian society. Up to now, there has been no BM pattern evaluation in Iranian employees. If we consider the normal range between 5th centile and 95th centile, according to our results, less than one BM per day suggests constipation while more than three BMs per day tends to diarrhea. The normal range was between one and three per day and the mode of BM frequency for workers was two per day. On the other hand, in a previous study in Iran on 995 subjects of the general population, the normal range had been determined between one and four, while more than four BM per day was considered as diarrhea. In that study, the majority of the population had two BMs per day similar to our study (22). In a Chinese study on the general population by Bassotti et al, the mean of BM frequency was calculated as one, and in this study less than one BM per day considered as a sign of constipation (19). In an Indian study by Panigrahi et al. on 1200 random subjects, the mean BM frequency of once a day and less than three times per week (according to western criteria) was considered as severe constipation while most people had two BMs per day (23). A Korean study by Jun et al. reported that most of the subjects with one BM per day as well as less than one BM per day were assumed as constipated (24). Our result agrees with the mentioned Asian surveys. Fiber consumption in Asian daily diet justifies increasing stool volume and diminished risk of constipation as compared to the Western population (10, 25, 26). For variables relating with BM frequency, we found a significant association between BM frequency and age (P-value=0.002), where BM declined with age. In the study by Panigrahi et al., the same association has also been confirmed (23). For marital and education status as well as exposure to chemical materials, we found a significant association with BM frequency (all P-values were less than 0.05). For marital and education status in the South Korean study, the same results have also been found (24). For chemical exposure, we did not find any documented study for comparison. For alcohol consumption, we did not find any significant result; except one study in Nigeria, which found uncertain significant results, there was no analogous study. For job features, the present study revealed that BM frequency had a statistically significant association with the work section of Auto factory (P-value <0.001); expectedly, this frequency in section A, which is not manual working, is less than in other sections (3). However, BM frequency did not have any relationship with work shift. For alcohol and smoking, we did not find any significant association, which is likely due to workers’ avoidance to answer these questions properly.

**Table 4:** Factors associated with functional constipation in Auto factory’s workers

Adjusted multivariate		Univariate		Without functional constipation	With functional constipation	Variable
P	OR (95% CI)	P	OR (95% CI)	N (%)	N (%)	
0.024^*^		0.043^*^				Age
-	1	-	1	345 (10.9)	22 (6.5)	20-30
0.006^*^	1.88 (1.20,3.03)	0.013^*^	1.75 (1.12,2.75)	2284 (72.3)	255 (74.8)	31-40
0.022^*^	1.91 (1.12,3.17)	0.011^*^	1.89 (1.14,3.12)	532 (16.8)	64 (18.8)	>=41
-	N	0.614				BMI**
		-	1	7 (0.2)	1 (0.3)	<18.5
		0.800	1.32 (0.16,10.75)	467 (14.8)	43 (12.8)	18.5-22.99
		0.341	0.85 (0.61,1.18)	2686 (85.0)	291 (86.9)	>=23
						Smoking
<0.001^*^	1.52 (1.20,1.93)	<0.001^*^	1.48 (1.18,1.85)	1026 (31.6)	141 (40.6)	Yes
	1	-	1	2217 (68.4)	206 (59.4)	No
-	N					Alcohol consumption
		0.791	0.94 (0.58,1.52)	189 (5.8)	19 (5.5)	Yes
		-	1	3054 (94.2)	328 (94.5)	No
						Exposure to chemical materials
0.175	1.31 (0.89,1.93)	<0.001^*^	1.80 (1.43,2.27)	854 (26.3)	136 (39.2)	Yes
	1		1	2389 (73.7)	211 (60.8)	No
						Work shift
	1	-	1	355 (11.1)	24 (7.0)	Only morning
0.406	0.80 (0.48,1.34)	0.025^*^	1.66 (1.08,2.55)	2850 (88.9)	320 (93.0)	Rotatory
	N					Marital status
		0.091	0.68 (0.43,1.05)	2948 (90.9)	325 (93.7)	Married
		-	1	295 (9.1)	22 (6.3)	Single or divorced
-	N	0.790				Education status
		-	1	123 (3.8)	11 (3.2)	Under diploma
		0.531	1.22 (0.65,2.30)	2003 (61.8)	219 (63.1)	Diploma
		0.632	1.17 (0.62,2.23)	1117 (34.4)	117 (33.7)	Upper diploma
<0.001^*^		<0.001^*^				Work section
	1	-	1	23 (0.7)	8 (2.3)	A^+^
0.023^*^	0.33 (0.12,0.87)	0.049^*^	0.44 (0.19,1.00)	712 (22.0)	108 (31.1)	P^++^
0.085	0.44 (0.18,1.10)	0.061	0.45 (0.19,1.02)	1268 (39.1)	198 (57.1)	M^+++^
<0.001^*^	0.06 (0.02,0.16)	<0.001^*^	0.06 (0.02,0.15)	971 (29.9)	21 (6.1)	B^++++^
<0.001^*^	0.12 (0.05,0.37)	<0.001^*^	0.13 (0.05,0.34)	269 (8.3)	12 (3.5)	P&P^+++++^

*: significant; **: body mass index; +: Administrative and supporting; ++: Paint; +++: Montag; ++++: Body; +++++: press & platform; N: not include in the multivariate logistic regression

We found a strong relationship between BM frequency and constipation (P-value < 0.001), where the prevalence of FC in the subjects was estimated as 9.7%. Our results are compatible with a South Korean population study with 9.2% prevalence (24). In the Iranian general population, various prevalence values of FC have been found ranging from 2.4% to 32.9% (27, 28). The different type of study population in our survey (young active male employees) vs. previous populations might be the reason of different results. 

In the present study, logistic regression model revealed that age, smoking, and job type may be introduced as constipation risk factors while the effect of work shift was not significant. For age and smoking, Kaboli et al. have reported similar results while they did not confirm the effect of BMI (29). For marital and education status, the study by Choung et al., as with our study, did not report any significant association (30). Male young active participants in our study might verify why work shift did not affect constipation; we know constipation is prevalent in sedentary, elderly, and female subjects (10). 

The strength of our study was that the study population consisted of a large working population with specific occupational conditions, including shift work and specific occupational segments. There have also been few studies of industry practitioners on gastrointestinal diseases such as constipation, and our study can offset this deficiency (31). In this study, we evaluated a great number of subjects; however, all subjects were men, as the factory rarely had a female worker. This point makes a limitation and no comparison was not made between men and women. Another limitation was related to the missing data. The questioners were instructed to complete the data entry forms as far as possible, but in some cases missing data were unavoidable and we had to delete high missing records from the dataset. Job- related stress was a factor whose influence was not assessed on the bowel habit and this is suggested for future study. 

 Our results revealed that the BM range for special population groups can be different in comparison with the general population. The occupational situation would cause varying BM patterns across the population. The job type may make difference in BM patterns. Rotatory shift and working in Administrative and Support (official) section as well as smoking may lead to heightened constipation’s risk. Consideration of the last facts may be helpful in constipation management.
